# Emotional prosody perception and production are linked in prelingually deaf children with cochlear implantsa)

**DOI:** 10.1121/10.0023996

**Published:** 2023-12-27

**Authors:** Monita Chatterjee, Ava Feller, Aditya M. Kulkarni, John J. Galvin

**Affiliations:** 1 Auditory Prostheses and Perception Laboratory, Center for Hearing Research, Boys Town National Research Hospital, 555 North 30th Street, Omaha, Nebraska 68131, USA; 2 House Institute Foundation, 1127 Wilshire Boulevard, Los Angeles, California 90017, USA monita.chatterjee@boystown.org, ava.feller@outlook.com, aditya.kulkarni@boystown.org, jgalvin@hifla.org

## Abstract

Links between perception and production of emotional prosody by children with cochlear implants (CIs) have not been extensively explored. In this study, production and perception of emotional prosody were measured in 20 prelingually deaf school-age children with CIs. All were implanted by the age of 3, and most by 18 months. Emotion identification was well-predicted by prosody productions in terms of voice pitch modulation and duration. This finding supports the idea that in prelingually deaf children with CIs, production of emotional prosody is associated with access to auditory cues that support the perception of emotional prosody.

## 1. Introduction

Due to the degraded spectro-temporal resolution provided by the device, cochlear implants (CIs) provide limited voice pitch information, an important acoustic cue to emotional prosody.^1^ As such, CI users' perception of emotional prosody is generally poorer than that of normally hearing (NH) listeners. Previous studies have shown deficits in emotional prosody identification in both postlingually deaf adults and prelingually deaf children with CIs, compared to NH listeners.^2–7^ Some studies have also shown that productions of emotional prosody are poorer in prelingually deaf children with CIs than in NH adults and children, as well as postlingually deaf adults with CIs.^8,9^ However, links between the perception and production of emotional prosody by prelingually deaf children with CIs are largely unknown. For example, previous studies^3,9^ have measured perception and production of emotional prosody in children with CIs; prosody production quality was rated by NH adults. Significant associations were observed between prosody perception by children with CIs and NH adult ratings of emotional prosody produced by the same children. While these studies provide some insight into the perceptual quality of pediatric CI users' emotional prosody production, they do not directly characterize the association between production and perception within the same group of children. Such information is important because it addresses scientific questions about how speech perception shapes speech production (in general, and specific to emotional prosody). Such information may also inform future CI signal processing schemes and rehabilitation strategies to improve emotion perception and/or production.

Auditory experience before cochlear implantation may differently affect emotional prosody perception and production. Perception of emotional prosody has been shown to be similar in postlingually deaf adult and prelingually deaf pediatric CI users.^5^ However, production of emotional prosody has been shown to be similar among postlingually deaf adult CI users, NH adults, and NH children; all of these three groups produced emotions that were more identifiable and exhibited larger acoustic contrasts than productions by prelingually deaf pediatric CI users.^8,9^ Thus, it is possible that previous acoustic hearing experience before implantation may benefit postlingually deaf adult CI users' emotional prosody production, but not their perception of emotional prosody. It is also possible that lack of acoustic hearing experience may negatively affect prelingually deaf pediatric CI users' production of emotional prosody.

To date, links between emotional prosody perception and the acoustic attributes of prosody production in prelingually deaf children with CIs have not been explored. It is unclear how the acoustic cues used for prosody perception relate to those associated with prosody production within this population. We hypothesize that perceptual access to the primary and secondary cues to emotional prosody is necessary to develop good emotional prosody production. As a first step toward testing this hypothesis, the objective of the present study was to examine links between emotional prosody perception and three aspects of emotional prosody productions by prelingually deaf school-age children with CIs: (i) mean voice pitch and variation in voice pitch, (ii) intensity, and (iii) duration. As in previous studies,^8,9^ participants were asked to read a list of 20 simple emotion-neutral sentences (e.g., *This is it; She is here; It is snowing again*) in a happy or a sad way, and the recorded productions were subjected to acoustic analysis. We hypothesized that emotional prosody perception would be correlated with the degree of acoustic contrast between happy and sad productions in terms of voice pitch, duration, and intensity, as well as participants' duration of CI device experience (i.e., hearing age). We predicted that larger contrasts in voice pitch, duration, and intensity would be associated with better emotion recognition performance. Early cochlear implantation provides pediatric CI recipients with auditory input during a period when the brain is maximally adaptive. We predicted that earlier cochlear implantation would be associated with larger acoustic contrasts in prosody production. However, based on previous work,^10^ we did not expect to observe such an advantage in prosody perception.

Emotional prosody perception was measured using stimulus sets of five uncontroversial emotions (happy, angry, neutral, sad, scared). Stimuli were produced in an exaggerated (child-directed) or an attenuated (adult-directed) manner. Using the same stimuli, a previous study^10^ with pediatric CI users showed that identification was better with exaggerated than with attenuated prosody. Exaggerated prosody was also associated with less inter-subject variability.^10^ Stronger links were expected between emotion production and perception of attenuated than of exaggerated prosody, as acoustic contrasts with attenuated prosody are weaker.^10^

## 2. Methods

Twenty prelingually deaf children with CIs (Table 1), aged 6–18 years (mean: 11.6 ± 4.4), with age at implantation ranging from 0.8 to 3.0 years (mean: 1.4 ± 0.52), and with duration of CI device experience (i.e., hearing age) ranging from 5.0 to 17.3 years (mean: 10.2 ± 4.1) were evaluated. None had usable acoustic hearing at birth, and all were native speakers of American English. None of the participants had any comorbidities associated with etiology of deafness, and all were otherwise healthy. None of the participants had diagnoses of cognitive or other impairment, and none were diagnosed with autism or autism spectrum disorder. Bilateral CI users were tested only while listening with their earlier-implanted CI. All participants were primarily oral communicators. Informed consent was obtained from parents/guardians, and informed assent was obtained from the participants. The work was conducted with approval from Boys Town National Research Hospital's Institutional Review Board (Protocol No. 11-24-XP).

**Table 1.** Demographic information for pediatric CI participants. The asterisks indicate participants who were simultaneously implanted; the remaining participants were sequentially implanted. The age at CI and hearing age are for the CI ear tested only.

Participant	Age at testing (years)	CI ear tested	Age at CI (years)	Hearing age (years)	Etiology of deafness	Ear(s) implanted	Device type
S1	11.6	Right	1.5	10.2	Pendred syndrome	Right	Advanced Bionics
S2	6.0	Right	1.0	5.0	Usher syndrome	Both	Advanced Bionics
S3	18.7	Left	1.4	17.3	Unknown	Both	Advanced Bionics
S4	8.5	Right	1.0	7.5	Unknown	Both	Advanced Bionics
S5	12.7	Left	1.5	11.3	Unknown	Both	Advanced Bionics
S6	16.3	Right	1.3	15.0	Auditory neuropathy	Both	Med-El
S7	18.5	Left	1.5	17.0	Unknown	Both	Advanced Bionics
S8	7.9	Right	1.2	6.7	Meningitis	Both	Cochlear Limited
S9	14.0	Right	2.2	11.8	Unknown	Both	Advanced Bionics
S10	17.4	Right	1.3	16.1	Unknown	Both; left CI not used	Advanced Bionics
S11	14.4	Right	1.2	13.2	Unknown	Both	Cochlear Limited
S12	10.8	Right	1.1	9.6	Connexin 26 mutation	Both	Advanced Bionics
S13	6.5	Right	0.8	5.7	Connexin 26 mutation	Both	Med-El
S14	7.8	Right	0.9	6.9	Unknown	Both	Advanced Bionics
S15	8.2	Right	1.3	6.9	Unknown	Both	Med-El
S16^*^	9.1	Right	1.7	7.4	Unknown	Both	Advanced Bionics
S17^*^	10.2	Right	1.1	9.1	Unknown	Both	Advanced Bionics
S18^*^	7.2	Right	1.9	5.3	Unknown	Both	Advanced Bionics
S19^*^	18.2	Left	3.0	15.2	Unknown	Both	Advanced Bionics
S20^*^	7.9	Right	1.0	6.9	Unknown	Both	Advanced Bionics

### 2.1 Tasks

#### 2.1.1 Emotional prosody perception

Stimuli were identical to those described in previous studies^5,11,12^ and consisted of recordings of 12 semantically neutral sentences produced according to five emotional targets: happy, angry, sad, neutral, scared. Stimuli were produced with exaggerated prosody by an adult female and male talker and with attenuated prosody by a different adult female and male talker. The stimulus set for each talker consisted of 60 stimuli (12 sentences × 5 emotions). Testing was blocked by talker, and test blocks were randomized within and across participants. Testing was conducted in a sound field inside a double-walled sound booth, with the listener directly facing a single loudspeaker 3 feet away. For each talker, the mean presentation level across all stimuli was 65 dBA in terms of root mean square (rms) amplitude; however, intensity differences were preserved among the stimuli, which varied substantially across the emotion targets. During testing, a stimulus was randomly selected and presented, and participants were asked to indicate the emotional intent by clicking on one of five response buttons shown on a computer screen. The buttons had cartoon faces illustrating the five emotions along with a text label. For each talker, accuracy was computed in terms of percent correct (chance level was 20% correct). Scores were averaged across the male and female talkers for the exaggerated and attenuated prosody.

#### 2.1.2 Emotional prosody production

As in previous studies,^8,9^ happy and sad emotions were selected for production because these two emotions are highly contrastive, both acoustically and conceptually. Young children with CIs have been shown to identify these two emotions as well as their NH peers in a facial emotion identification task,^3^ suggesting that the concepts of these emotions are established in school-age children with CIs.

As in previous studies,^8,9^ a list of 20 simple, semantically emotion-neutral sentences was created to record emotional prosody production (e.g., *This is it; She is here; It is snowing again*). Participants were asked to read the sentences aloud in a happy and a sad way, with no training or exemplars provided, and with no specific feedback other than general encouragement. All participants were able to read the sentences without difficulty. Participants were encouraged to practice before recording started and were asked to indicate when they were ready to begin recording. Recordings were made using a microphone (AKG 1000, Harman International, Stamford, CT) connected to an audio interface (Edirol UA-25, Roland, Hamamatsu, Japan). Three sets of recordings of the 20 sentences were made for each emotion. Typically, the second of the three sets was used for acoustic analyses. If a particular recording was noisy or difficult to analyze for some reason, a corresponding recording was used from the first or third set.

Acoustic analyses of the happy and sad recordings were conducted by A.F. using Praat^13^ software (version 6.3.09), with checks by the first author for the pitch range and settings for each sentence. The mean fundamental frequency (F0) of the F0 contour (overall pitch height), the standard deviation in F0 across the contour (variance in pitch), the mean intensity, and the duration were obtained for each recording. In the NH population (both school-age children and adults), the mean F0, F0 standard deviation, and intensity are typically greater for happy than for sad productions, and duration is shorter for happy than for sad productions.^9^ The ratio of the values between the happy and sad productions (i.e., “contrast”) was calculated for each sentence in terms of the mean F0, the standard deviation of F0 within the contour, and the duration. The dB difference between the mean intensities for the happy and sad productions was also calculated for each sentence. For each participant, the average of these ratios and intensity differences was calculated across all productions.

#### 2.1.3 Statistical analyses

Statistical analyses were performed using R version 4.0.4.^14^ Multiple linear regression analyses were conducted to examine the relationship between perception and production of emotional prosody. Variables included contrasts between happy and sad productions in terms of mean F0, the variance of F0, overall duration, and mean intensity, as well as age at implantation and hearing age (the difference between age at testing and age implantation). A hierarchical approach was taken to determine inclusion of variables in the final model. Goodness of fit for the models was ensured by examining the distributions of model residuals, the adjusted *r*^2^ values, and significance levels. More complex models were compared with simpler models via a likelihood ratio test by using the *anova* function in the *car* package in R.^15^

## 3. Results

In exploratory analyses, hearing age and age at implantation were compared to the acoustic contrasts between the happy and sad productions, as well as to perception of exaggerated or attenuated emotional prosody. Linear regression analysis showed that hearing age was significantly correlated with the F0 variance contrast [beta (estimated coefficient) = 0.122, adjusted *r*^2^ = 0.37, *p* = 0.003] and intensity difference (beta = 0.694, adjusted *r*^2^ = 0.55, *p* < 0.001), but not with the duration ratio. Hearing age was also significantly correlated with perception of attenuated emotional prosody (beta = 2.02, adjusted *r*^2^ = 0.24, *p* = 0.018), but not with perception of exaggerated prosody. However, hearing age was not a significant predictor of perception of attenuated or exaggerated emotional prosody in multiple regression analyses when combined with other variables (see below). Age at implantation was not significantly correlated with contrasts in F0 variance, duration, or intensity or with perception of attenuated or exaggerated emotional prosody The intensity difference was significantly correlated with the F0 variance contrast (beta = 3.31, adjusted *r*^2^ = 0.46, *p* < 0.001), but the duration ratio was not correlated with either the F0 variance contrast or the intensity difference.

### 3.1 Acoustic contrasts in the production vs perception of exaggerated emotional prosody

**Fig. 1.**
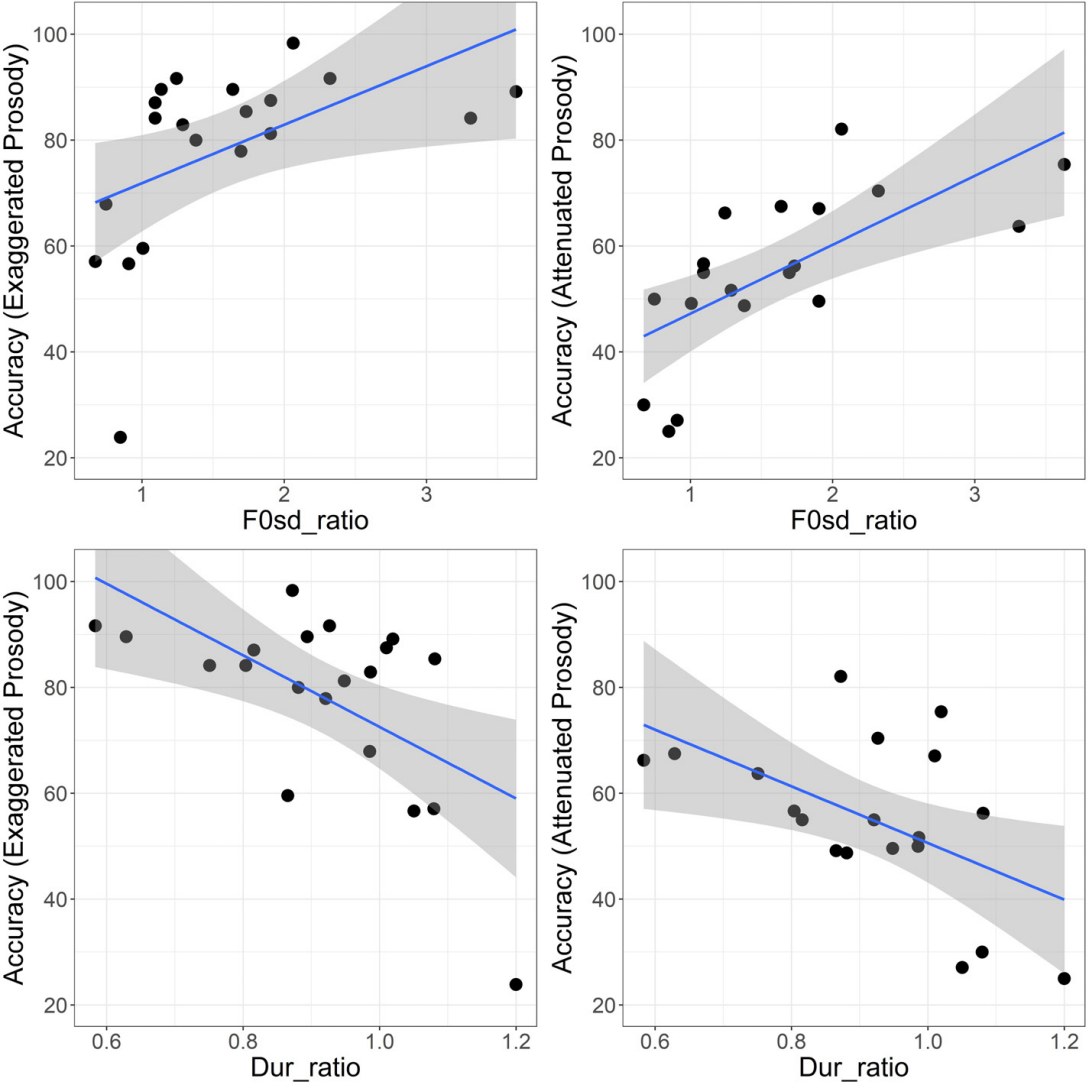
Identification of exaggerated (left) or attenuated prosody (right) as a function of the ratio between happy and sad productions from the same participants in terms of F0 standard deviation (F0sd_ratio) (top) or duration (Dur_ratio) (bottom). The solid lines show the fit, and the shaded areas show the 95% confidence intervals from a linear regression model.

The top left panel of Fig. 1 shows the accuracy in perception of exaggerated emotional prosody as a function of the contrast of the F0 variance between the happy and sad productions. As exploratory linear regression analysis showed no significant relationship between the perception of exaggerated prosody and the mean F0 contrast, the mean F0 contrast was excluded from the subsequent multiple linear regression analyses. However, F0 variation contrast was significantly correlated with identification of exaggerated prosody (beta = 11.05, adjusted *r*^2^ = 0.21; *p* = 0.024). Including the duration contrast significantly improved model fit. When the model included both the F0 variance and the duration contrasts, the estimated coefficient values were 9.52 for the F0 variance contrast [*t*(3.70) = 2.57, *p* = 0.020] and −61.05 for the duration contrast [*t*(19.47) = −3.14, *p* = 0.006]. The adjusted *r*^2^ increased from 0.21 with only the F0 variance contrast to 0.47 when both the F0 variance and duration contrasts were included in the model. Including age at implantation or hearing age did not improve the model fit. Because the variance inflation factor was <1.7 for all predictors, including hearing age was not a concern for collinearity, despite its significant correlation with the F0 variance contrast (see above). The intensity difference had also shown a significant correlation with the F0 variance contrast (indicated previously). Including the intensity difference in the model resulted in a variance inflation factor of 2.03, suggesting a mild concern regarding collinearity. However, as this variable did not significantly improve the model, it was not included in the final model.

*Post hoc* analysis indicated that statistical power with the 20 participants was only 0.70 (based on adjusted *r*^2^), possibly because the scores obtained with the exaggerated prosody were generally high (i.e., the data may have included ceiling effects).

### 3.2 Acoustic contrasts in production vs perception of attenuated emotional prosody

As with exaggerated emotional prosody, exploratory linear regression analysis showed no significant correlation between the mean F0 contrast and perception of attenuated prosody. Accordingly, the mean F0 contrast was excluded from subsequent analyses. However, the F0 variance contrast was significantly correlated with perception of attenuated prosody [beta = 13.02, adjusted *r*^2^ = 0.43; *F*(1,17) = 14.43, *p* = 0.001]. Including both the F0 variance and duration contrasts significantly improved model fit. In the model that included both the F0 variance and duration contrasts, the estimated coefficient values were 11.83 for the F0 variance contrast [*t*(2.84) = 4.17, *p* = 0.001] and –45.07 for the duration contrast [*t*(14.82) = –3.04, *p* = 0.008]. The adjusted *r*^2^ increased from 0.43 with only the F0 variance contrast to 0.61 when both the F0 variance and duration contrasts were included in the model. Including the intensity contrast, age at implantation, or hearing age did not improve the model fit. Again, the variance inflation factor was <1.7, so including hearing age in the model was not a concern for collinearity. As with the model for exaggerated prosody, including the intensity difference resulted in a variance inflation factor of 2.003, consistent with the observed correlation with the F0 variance contrast, but as this variable was not included in the final model, the collinearity concern does not arise. *Post hoc* power analysis of the final model using the adjusted *r*^2^ value showed a power of 0.82 with the 20 participants.

Because the final models for perception of exaggerated and attenuated speech share predictor variables, Bonferroni correction for multiple comparisons was applied to the model fits (adjusted alpha = 0.025). Significance for both models persisted after Bonferroni correction for all model fits.

## 4. Discussion

The present results suggest a significant relationship between emotional prosody perception and the degree of acoustic contrast between happy and sad productions in prelingually deaf children with CIs. Similar patterns were observed for perception of both exaggerated and attenuated prosody. In both cases, the F0 variance contrast (the difference in voice pitch modulation between happy and sad productions) was significantly correlated with emotion identification scores. The ratio of F0 variation between happy and sad productions should be >1.0, as the voices are typically more modulated for happy than for sad emotions. Most participants exhibited values >1.0, suggesting that children with CIs are able to produce the relative happy and sad voice pitch modulations in the correct direction. The ratio of duration between happy and sad productions should be <1.0, as happy speech is typically produced faster than sad speech. Again, most participants exhibited ratios <1.0, again suggesting that children with CIs are able to produce the relative happy and sad durations in the correct direction. When combined, the contrast in F0 variance and duration accounted for 47% and 61% of the variance in identification of exaggerated and attenuated prosody, respectively. The effects were robust, with significance of the individual predictors and the overall models persisting after Bonferroni correction for multiple comparisons.

The results also showed a significant positive relationship between the participants' duration of device experience (hearing age) and emotional prosody identification scores, consistent with previous findings.^10^ In addition, we found a positive relationship between hearing age and the contrast between happy and sad productions in two of the three acoustic domains investigated: F0 variance and intensity. This suggests some scope for developmental plasticity in emotional prosody productions by children with CIs. However, hearing age was not a significant predictor of emotion recognition when the other predictors were included, suggesting that it may not contribute to the relationship between perception and production of emotional prosody.

While correlation does not imply causation, it is reasonable to hypothesize that perception mediates development of production in children. Thus, acoustic characteristics of native languages are imprinted in infants' babble,^16,17^ while children with significant untreated hearing loss may develop speech with specific acoustic characteristics.^18^ The perception-production link shown in the present study suggests that even with the degraded acoustic cues associated with electric hearing, prelingually deaf children with CIs are able to utilize what they hear to learn to express emotional prosody.

These findings have implications for the design of speech therapy protocols targeting prosodic expression of emotions in children with CIs. The present findings also suggest that the deficits in perception extend to deficits in production in the pediatric CI population, with further implications for overall reduced social communication, social isolation, and reduced quality of life. Thus, there is a critical need to develop clinical and technological diagnostic tools to assess and treat difficulties in prosodic communication by children with CIs. Given the importance of emotional communication for development in children, improving perception and production of emotional prosody will positively impact the lives of pediatric CI users.

## Data Availability

The data associated with this study are publicly available at the Open Science Framework (https://osf.io/z3qg6/).
